# Hypofractionated Radiation Therapy for Pain Relief of Patients With Spinal Metastasis: A Real‐World Analysis

**DOI:** 10.1002/cnr2.70451

**Published:** 2026-01-29

**Authors:** Lu Sun, Fan Luo, Yajuan Zhou, Xiyou Liu, Pingping Zhang, Dan Li, Yi Peng

**Affiliations:** ^1^ Department of Radiation Oncology, Hubei Cancer Hospital, Tongji Medical College Huazhong University of Science and Technology Wuhan China; ^2^ Department of Oncology The People's Hospital of Xiangzhou District Xiangyang Hubei China

**Keywords:** bone metastasis, hypofractionated radiation therapy, pain management, radiotherapy regimen

## Abstract

**Purpose:**

Spinal metastases may cause pain, neurological compromise, paraplegia, and limb movement disorders; their management requires a comprehensive approach. Alongside systemic anti‐tumor therapies, focal interventions such as radiotherapy, bone‐modifying agents, and surgery are crucial for slowing disease progression and managing pain in spinal metastases. However, substantial variations exist in radiotherapy regimens for spinal metastases. In this study, we aimed to investigate the safety and efficacy of hypofractionated radiation therapy (HFRT) regimens at our hospital, specifically to evaluate pain relief and incidence of re‐irradiation after HFRT.

**Methods:**

In this retrospective study, data from 58 patients diagnosed with spinal metastasis who received HFRT (4.5–10Gy * 3–7F) at our center between December 2017 and June 2022 were analyzed. All patients were followed up from the initiation of HFRT to either death or their last follow‐up visit. Degree of pain was assessed using the numeric rating scale (NRS) before and after 1 month of HFRT. A multivariate Cox regression model was established to identify the independent risk factors for prognostic analysis of spinal metastasis.

**Results:**

HFRT could effectively manage pain in patients with spinal metastasis. The pain scores were significantly decreased after HFRT (3.43 vs. 1.5, *p* < 0.001), with 84.5% patients experiencing improved pain relief 1 month after radiotherapy. No cases of radiation myelitis were observed during the follow‐up period. Furthermore, the incidence of re‐radiotherapy was significantly increased in patients with spinal metastases who received moderate HFRT (< 5 Gy/day) (*p* = 0.01, HR = 0.43).

**Conclusion:**

HFRT significantly reduced pain scores and reirradiation rates without increasing radiation myelitis incidence for spinal metastases.

## 1 Introduction

Spinal metastases commonly occur in patients with advanced lung, breast, kidney, and prostate cancers, representing approximately 30% of all bone metastases [1]. Skeletal‐related events (SREs) resulting from bone metastases, including bone pain, fractures, spinal cord compression, and hypercalcemia, are associated with shorter survival and poorer quality of life [2, 3]. Achieving a complete cure for patients diagnosed with spinal metastasis of a malignant tumor is challenging. The treatment of spinal metastases remains suboptimal, focusing on preventing disease progression and alleviating pain rather than seeking a cure. Current treatment modalities for spinal metastases include anticancer drugs, bisphosphonates, surgery, and radiotherapy. Radiotherapy is a well‐established and effective modality for relieving pain and improving the quality of life in patients with bone metastasis [4].

Conventional external beam radiation therapy (cEBRT) is considered the standard treatment for spinal metastases [5]. It has been shown to yield an overall pain response (OR) and complete pain response (CR) rates of approximately 70% and 25%, respectively [6, 7]. In contrast to non‐spinal metastases, lower total doses and lower doses per fraction may be required to avoid damaging neurological structures [8]. Radiation therapy for spinal metastases demands advanced techniques and equipment due to dose limits on the spinal cord, ensuring a rapid dose drop‐off from the treatment field to outside of the treatment field [9]. Recently, many researchers have begun to explore new radiotherapy patterns. Numerous studies and clinical trials have confirmed the efficacy of 8 Gy/1F, 20 Gy/5F, and 30 Gy/10F radiation doses [10]. A randomized Phase III study by the Radiation Therapy Oncology Group (RTOG) demonstrated a favorable pain relief effect using stereotactic radiosurgery compared to cEBRT for spinal metastasis, with no significant difference in the incidence of acute or late adverse effects between them [11]. Similar conclusions have been confirmed in other studies; HFRT demonstrated a statistically significant improvement in complete pain response compared to cEBRT, with similar local progression and overall survival (OS) and lower skin reactions [12, 13, 14, 15]. However, it is worth noting that HFRT usually requires hospitalization for 3–10 days, while EBRT takes 2 to 3 weeks or even longer. In conclusion, pain control was more favorable with HFRT than cEBRT, and HFRT also resulted in a shorter treatment duration and more convenience. According to guidelines of the American Urological Association (AUA), American Society of Clinical Oncology (ASCO), and the American Society for Radiation Oncology (ASTRO), HFRT can be classified into two types: moderate‐HFRT (m‐HFRT), delivering 2.4–3.4 Gy/daily fractions and extreme HFRT (e‐HFRT), delivering > 5 Gy/daily fractions [16]. To date, there is no consensus regarding the optimal radiation dose and fraction.

Data on HFRT regimens for spinal metastases remain limited. Small single‐institution studies have found that HFRT is well‐tolerated, with acceptable rates of toxicity, disease control, and OS [16]. Additional evidence, along with further prognostic and outcome data, is required to thoroughly assess the effectiveness of HFRT in patients with bone metastases. Hence, this retrospective study was conducted in patients with bone metastases who were treated with HFRT. By examining the outcomes of patients treated with HFRT, the study seeks to provide insights into the effectiveness of this modality in managing bone metastases. This includes assessing pain relief, disease control, and OS, offering clinicians a better understanding of HFRT's performance in real‐world scenarios. The study aims to explore different regimens and their associated outcomes and contribute to the ongoing discussion about standardizing HFRT protocols for bone metastases.

## 2 Material and Methods

In this retrospective study, we analyzed data collected from patients diagnosed with spinal metastasis who received radiation therapy at our center between December 2017 and June 2022. All patients were followed up from the initiation of HFRT to death or the last follow‐up visit. The follow‐up time was 0.3–55 months, and the median follow‐up time was 8.5 months. OS was defined as the time period from the start of HFRT to the last follow‐up or death. Patient data were extracted from the hospital's electronic medical records, and the inclusion criteria were as follows: (1) diagnosis of spinal metastasis, (2) HFRT provided to patients at the Department of Radiation Oncology at Hubei Cancer Hospital, (3) no serious heart, liver, kidney, and blood system, and other important organ diseases and dysfunctions, and (4) a complete patient history. The exclusion criteria were as follows: (1) the spine as the primary site of treatment, (2) underwent history of en bloc spondylectomy, vertebroplasty, or kyphoplasty, and (3) received history of RT at the same anatomical site, which may increase the risk for radiation myelopathy, and (4) the patient experiences pain in areas other than spinal metastases. Clinical data, encompassing age, sex, serum calcium ions, serum phosphorus ions, alkaline phosphatase (ALP), use of painkillers and bisphosphonates, tumor histology, subtype, number of metastases, and The Eastern Cooperative Oncology Group (ECOG) score were collected. Patients were assessed for pain using the numeric rating scale (NRS) pre‐RT and 1 month after completing RT under the standardized use of painkillers. Variables with no missing value and having no multicollinearity were included in bivariate analyses. Statistical analysis was performed using SPSS Version 27. Intergroup comparisons were conducted using the independent‐samples *t*‐test, whereas intragroup comparisons were conducted using the paired *t*‐test. The independent variables were tested in a multivariate model by the stepwise forward method. The independent variables were assessed using uni‐Cox and multi‐Cox regression analyses. Statistical significance was set at a *p* value < 0.05.

## 3 Results

### 3.1 Baseline Characteristics of the Study Population

Between December 2017 and June 2022, 58 patients with spinal metastases underwent HFRT at our center. In this study, the total dose is 18–40 Gy. More than half of the patients received radiotherapy at a dose of 30 Gy in 6 fractions or 35 Gy in 7 fractions. The patient and general characteristics are detailed in Table 1. The median age at the time of treatment was 59.1 years (range, 14–91 years), and two‐thirds (*n* = 38) of the patients were male individuals. Approximately 65.5% of patients were ≤ 65 years of age (*n* = 38). The primary tumor in 67.2% of these patients was lung cancer, followed by liver, breast, and prostate cancers. Distant metastases to other sites were observed in 40 patients (68.9%), with the lungs being the most common metastatic site, followed by the liver and brain. All but one patient experienced pain, with a mean pain score of 3.43 on the NRS before treatment. More than 40% of the patients were rated as having moderate or strong pain.

**TABLE 1:** Characteristics of patients with bone metastasis assessed between December 2017 and June 2022, median follow‐up after discharge was 111 days (IQR: 42–165 days).

Characteristics	Total	m‐HFRT	e‐HFRT	*p*
Age, years				0.7503
≤ 65	38	10	28	
> 65	20	4	16	
Sex				0.1064
Male	38	12	26	
Female	20	2	18	
Primary tumor				0.9796
Breast cancer	5	1	4	
Prostate cancer	4	1	3	
Liver cancer	6	2	4	
Lung cancer	24	6	18	
Other	19	4	15	
Pain score				0.3560
0–3	33	10	23	
4–6	22	4	18	
7–10	3	0	3	
Distant metastases at baseline				0.4696
Lung	18	3	16	
Distant node metastases	5	2	3	
Brain	9	2	7	
Liver	13	2	11	
Other	8	3	5	
None	20	2	18	
ECOG				0.6208
0–2	33	12	31	
3–4	5	2	3	

### 3.2 Pain Response by Treatment

The patient characteristics related to pain are summarized in Table 2. The median baseline pain score (worst pain in the last 72 h) was 3.43 on a scale of 0 to 8. Patients with pain (*n* = 57) were grouped into the following categories: neuropathic (*n* = 2), somatic (*n* = 50), and mixed pain (*n* = 7). Among the 58 patients, 16 (27.6%) presented with documented radiographic cord compression. At baseline, 84.4% of the patients required painkillers, with NSAIDs effectively relieving pain in two patients. Among the remaining patients, 15.5% (*n* = 9) used weak opioid analgesics, and 65.5% (*n* = 38) used strong opioids as their highest level of pain medicine. Five patients required combination therapy for pain relief. Nausea, vomiting, constipation, and dizziness were the most common adverse reactions to opioid analgesics. Fourteen patients experienced adverse effects of painkillers.

**TABLE 2:** Correlation of patient characteristics with pain scores.

Medications at baseline	
None	9
NSAID	2
Weak opioid analgesics	9
Strong opioid analgesics	38
Pain characteristics	
Somatic	50
Neuropathic components	2
Mixed pain	5
Spinal compression	
Yes	16
No	42

**FIGURE 1:**
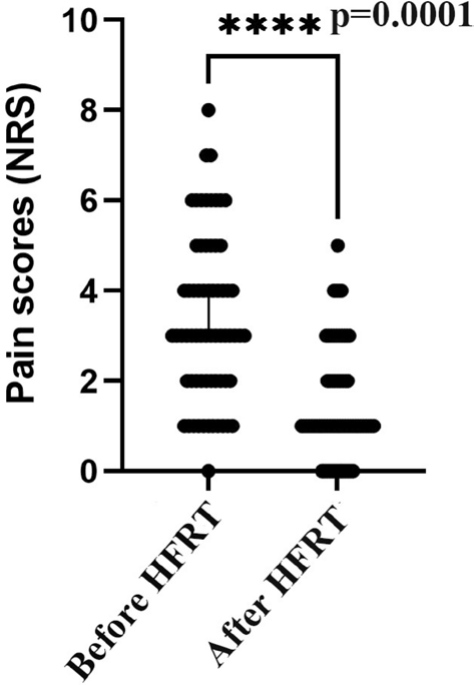
Pain intensity (in NRS) before and after HFRT.

The overall treatment response was evaluated 1 month after HFRT. Five patients achieved complete response (CR) at the first follow‐up after radiotherapy. Approximately 93% (*n* = 54) of patients experienced significant pain relief, with none reporting worsening conditions. Out of the 58 patients, 49 achieved improvement in pain relief after radiotherapy. Five patients experienced no significant change in their level of pain. The median NRS score of the 58 patients presenting with pain was 1.5 (range, 0–4) after HFRT. The reduction in pain score was statistically significant (Figure 1, *p* < 0.001).

### 3.3 Survival and Bone Outcomes

**FIGURE 2:**
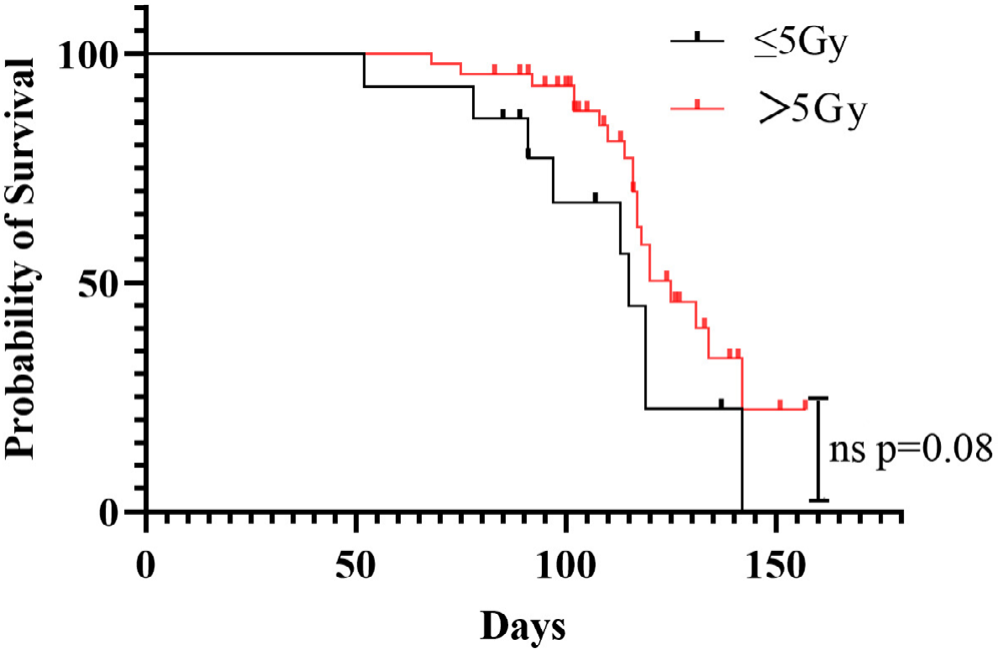
OS between the high‐dose and low‐dose groups.

Among the 58 patients who received HFRT, 8 (13.7%) experienced novel bone lesions, and 9 (15.5%) experienced competing events of death. Of those nine patients, four succumbed to respiratory failure, three to cancer cachexia, one to upper gastrointestinal bleeding, and one to hepatic failure. Factors that may be related to reirradiation were included in the correlational analyses. Multivariate analysis suggested that the fraction dose, ECOG score, and serum calcium level were independent factors influencing the occurrence of reirradiation (Table 3). The use of bisphosphonates, total dose, spinal compression, and ALP levels did not correlate with bone outcomes in patients after HFRT. In particular, a high fraction dose (> 5 Gy) was negatively correlated with reirradiation incidence, whereas calcium level and ECOG score were positively correlated with reirradiation incidence (Table 4). Additionally, no significant correlations were observed between OS and age, sex, ALP level, total dose, local control (status), spinal compression, pain relief, bisphosphonates, serum phosphorus, neutrophils count, neutrophil count/lymphocyte count ratio, platelet count/lymphocyte count ratio, or hemoglobin. Similarly, there was not much difference in OS between the high‐dose and low‐dose groups (Figure 2).

**TABLE 3:** Univariate analyses of the pain relief of patients.

Variables	Categories	Relieved (*n*)	Not relieved (*n*)	*p*
Fraction dose	≤ 5GY	14	0	0.3222
	> 5GY	39	5	
Fraction	< 5F	17	2	0.6330
	≥ 5F	41	3	
Total RT dose	≤ 30GY	30	3	0.988
	> 30GY	28	2	
Bisphosphonates	No	24	1	0.6399
	Yes	34	4	
ECOG	0–1	32	2	0.6544
	2–4	26	3	
ALP	Abnormal	20	1	0.6439
	Normal	33	4	
Serum calcium	Abnormal	27	0	0.0547
	Normal (2.25–2.58 mmol/L)	26	5	
Serum phosphorus	Abnormal	12	1	0.999
	Normal (0.87–1.45 mmol/L)	41	4	
Spine compression	Yes	7	1	0.5376
	No	46	4	

Abbreviations: ALP, alkaline phosphatase; *B*, beta (regression coefficients); ECOG, Eastern Cooperative Oncology Group performance status; *p*, *p* value; RT, radiation therapy.

**TABLE 4:** Multivariate analyses of the irradiation of patients.

Variables	Categories	*n*	*B*	*p*
Fraction dose	≤ 5GY	14	6.118	0.01
	> 5GY	44		
Fraction	< 5F	17	2.527	0.064
	≥ 5F	41		
Total RT dose	≤ 30GY	30	−3.448	0.065
	> 30GY	28		
Bisphosphonates	No	24	2.859	0.091
	Yes	34		
ECOG	0–1	32	−2.244	0.028
	2–4	26		
ALP	Abnormal	21	1.504	0.415
	Normal	37		
Serum calcium	Abnormal	27	−3.743	0.022
	Normal (2.25–2.58 mmol/L)	31		
Serum phosphorus	Abnormal	13	−0.832	0.479
	Normal (0.87–1.45 mmol/L)	45		
Spine compression	Yes	8	−1.221	0.540
	No	50		

Abbreviations: ALP, alkaline phosphatase; *B*, beta (regression coefficients); ECOG, Eastern Cooperative Oncology Group performance status; *p*, *p* value; RT, radiation therapy.

## 4 Discussion

Although almost all types of cancer can spread to the bones, the most common tumors associated with spinal metastases are breast, prostate, thyroid, lung, and kidney cancers. The treatment options for spinal metastases are limited, and generally, the treatment is palliative. Comprehensive therapies, including chemotherapy, surgery, radiotherapy, and bisphosphonates, can reduce SREs and improve the quality of life in patients with spinal metastases. Previous studies have suggested that radiotherapy is the standard approach for treating symptomatic spinal metastases. Emerging evidence suggests that preventive irradiation is necessary for asymptomatic high‐risk spinal metastases. Preventive radiation can reduce the incidence of SREs with controllable toxicity [17]. Radiotherapy is typically the mainstay of treatment for spinal metastases.

For spine metastases, the effectiveness of HFRT is highly controversial with conflicting results compared with cEBRT [18]. In recent meta‐analysis, HFRT include its potential to provide long‐lasting and persistent pain relief but a higher fracture risk linked with a target volume [19, 20]. It seems that the higher BED may bring a better therapeutic effect. However, this was not the case. Xing Song et al. observed that excessive elevation of biological equivalent dosage (BED) introduces the risk of diminishing the analgesic effect of HFRT. But, HFRT may has the analgesic advantage under the same BED [21]. Patients with bone metastasis require frequent opioid treatment. Increasing evidence has shown that high‐dose opioid exposure may increase the risk of gastrointestinal events, infections, falls and fractures, and cardiovascular events [22]. HFRT is associated with a quicker and improved pain response and a reduced dose of morphine 3 months after treatment [23, 24]. This is another potential advantage of HFRT for patients with spinal metastases, including reducing medical expenses and the economic burden on patients [25].

A study by Sakr et al. at a single center suggested that an HFRT regimen of 27 Gy/3 fractions provides a pain‐relieving effect comparable to that of the standard fractionation schedule of 20 Gy/5 F, with the additional benefit of immediate pain relief [26]. However, in a study in Europe, they found that no difference between SRS (16–18 Gy in 1 fraction) treatment in improving pain relief and local tumor control compared to cEBRT [26]. A meta‐analysis by Kei Ito also considered that there is no significant difference between HFRT and cEBRT in the incidence of severe adverse effects and health‐related quality of life outcomes [27]. In our study, HFRT provided pain relief to 92% of the patients, with up to 10% achieving CR. This response rate is higher than the 62.6%–77% reported for bone metastasis after radiotherapy [28, 29]. Radiation myelitis and **v**ertebral fractures were not observed during the follow‐up. No myelosuppression was observed during radiotherapy.

Van de Ven et al. observed a lower incidence of reirradiation with HFRT compared to conventional radiotherapy [30, 31]. Additionally, Zhao et al. observed in non‐small cell lung cancer mouse models that 12.4 Gy/2 F resulted in longer survival times than other fractionations with the same biological equivalent dose of 20 Gy. This observation may be attributed to the induction of more intratumoral infiltrating T cells after HFRT, enhancing the body's anti‐tumor immune response, especially with a regimen of 12.4 Gy/2 F [31]. Due to the success of the combination of immunotherapy and RT, interest in this phenomenon has recently regained momentum. However, the internal mechanism of action is not fully understood. In clinical studies, HFRT combined with immunotherapy exerted superior anti‐tumor efficacy compared to that of c‐CBRT in solid tumors. Patients with melanoma who received HFRT combined with immunotherapy had a higher 3‐year OS than those treated with cEBRT alone (37.3% vs. 8.6%; *p* < 0.0001) [32]. In the current study, the fraction dose was positively correlated with preventing the incidence of reirradiation. Specifically, fractions > 5 Gy had a significant advantage in inhibiting the occurrence of reirradiation, potentially associated with the activation of endogenous anti‐tumor immunity. This hypothesis still requires further verification.

Higher calcium levels are typically observed in patients with osteolytic lesions. Patients with spinal metastases rarely experience hypercalcemia‐related symptoms. We found that high calcium level was an independent risk factor for the occurrence of reirradiation. This is consistent with the conclusions of previous studies [33].

Our study has many limitations that warrant improvement. First, it was a retrospective study rather than a prospective, controlled, randomized study, introducing the possibility of bias from missing data and inaccuracies that may have affected our results. Second, pain rating was subjective, and the evaluation criteria were not strictly defined. Therefore, several factors could interfere with the final results. Finally, the small number of patients and low number of primary events may have limited the statistical analyses. Despite these limitations, this study provides valuable insights into the use of HFRT for treating spinal metastases.

## 5 Conclusion

In conclusion, HFRT is an effective method for alleviating bone pain associated with spinal metastasis. Concurrent HFRT does not increase the incidence of radiation‐induced myelitis. e‐HFRT has a lower incidence rate of re‐irradiation than m‐HFRT. These findings highlight the potential benefits of HFRT, particularly in combination with immunotherapy, paving the way for further exploration and refinement of treatment strategies for spinal metastases.

## Author Contributions

The manuscript was written through contributions of all authors. Conception, overall monitoring and final approval of the article were done by Yi Peng and Yajuan Zhou. Target delineation was done by Pingping Zhang and Xiyou Liu. The design of radiotherapy plans was finished by Dan Li. Data analysis and first draft writing was done by Lu Sun and Fan Luo.

## Funding

This work was supported by the Wuhan Young & Middle‐Aged Medical Backbone Training Program and the Beijing Science Innovation Medical Development Fund (KC2023‐JX‐0186‐PQ008).

## Ethics Statement

Ethics approval was obtained by the Ethics Committee of Hubei Cancer Hospital (No. LLHBCH2024YN‐029). Every subject involved in the study gave informed consent to participate in the study, which designated this study as exempt research, waiving the requirement of informed consent.

## Consent

The authors have nothing to report.

## Conflicts of Interest

The authors declare no conflicts of interest.

## Data Availability

The data that support the findings of this study are available upon request to the corresponding author.
